# Effect of Plasmonic Ag Nanoparticles on Emission Properties of Planar GaN Nanowires

**DOI:** 10.3390/nano13081421

**Published:** 2023-04-20

**Authors:** Galia Pozina, Carl Hemmingsson, Natalia Abrikossova, Elizaveta I. Girshova, Erkki Lähderanta, Mikhail A. Kaliteevski

**Affiliations:** 1 Department of Physics, Chemistry and Biology, Linköping University, 58183 Linköping, Sweden; carl.hemmingsson@liu.se (C.H.);; 2 Department of Physics, LUT-University, 53850 Lappeenranta, Finland; ilinishna@gmail.com (E.I.G.); erkki.lahderanta@lut.fi (E.L.); 3 National Graphene Institute, The University of Manchester, Manchester M13 9PL, UK; mikhail.kaliteevski@manchester.ac.uk

**Keywords:** GaN planar nanowires, plasmonic silver nanoparticles, photoluminescence, Fröhlich resonance

## Abstract

The combination of plasmonic nanoparticles and semiconductor substrates changes the properties of hybrid structures that can be used for various applications in optoelectronics, photonics, and sensing. Structures formed by colloidal Ag nanoparticles (NPs) with a size of 60 nm and planar GaN nanowires (NWs) have been studied by optical spectroscopy. GaN NWs have been grown using selective-area metalorganic vapor phase epitaxy. A modification of the emission spectra of hybrid structures has been observed. In the vicinity of the Ag NPs, a new emission line appears at 3.36 eV. To explain the experimental results, a model considering the Fröhlich resonance approximation is suggested. The effective medium approach is used to describe the enhancement of emission features near the GaN band gap.

## 1. Introduction

III-nitride semiconductor nanostructures are attracting significant interest today due to a wide range of potential optoelectronic and nanophotonic applications such as light emitting diodes (LEDs) [1], water splitting [2], waveguides [3], microcavities [4,5], and sensors [6]. The manufacturing process usually involves the epitaxial growth of multilayers on a substrate with postgrowth lithography and processing, known as the so-called top-down paradigm [7]. An alternative bottom-up method [7] for the fabrication of III-N nanostructures includes a selective area epitaxy [8,9] or the growth techniques based on processes with the self-organized formation of nanostructures [10]. A promising method to produce nanostructures with controllable sizes and shapes is a selective area metalorganic vapor phase epitaxy (MOVPE), where a given pattern in a mask can be etched by a focused ion beam (FIB) [11]. One of the most important nanophotonic challenges is related to the design of nanostructures for the control and manipulation of emission properties. 

Previously, it had been shown that high-quality planar GaN nanowires grown using a bottom-up approach possess properties of optical microcavities where an enhancement of spontaneous emissions can be observed [12]. The search for new ways to modify optical spectra is an urgent task of nanophotonics which explains that in recent years, new types of resonators have been actively studied, for example, structures with a Tamm plasmon [13] and meso-resonators [14]. Another method for affecting emission properties in semiconductor materials is to use plasmonic nanoparticles (NPs) that can interact with light and can significantly enhance the excitation of semiconductors due to the localized plasmon resonance [15]. In addition, plasmonic properties can vary depending on the size and shape of NPs and the metal used [16]. Plasmonic effects have been used for many applications such as in photovoltaics [17], biosensing [18], and LEDs [19]. The performance of GaN-based optoelectronic devices has been improved using silver NPs with a resonance frequency from the near-UV to visible regions depending on the NP size [20].

The combination of unique properties exhibited by semiconductor nanowires (NWs) and the interesting properties of metal NPs enables the creation of hybrid nanostructures with a high degree of design freedom. For example, depositing Ag NPs on Si NWs has been shown to enhance the photocurrent significantly compared to uncoated samples [21,22]. Additionally, there have been examples of luminescence enhancement in semiconductor NWs, such as a sixfold increase in luminescence observed in ZnO NWs when decorated with metal NPs [23]. Studies have shown that by depositing Ag NPs on the top of GaN/InGaN multiple quantum wells, LED structures can improve the internal quantum efficiency (IQE) by coupling optical transitions in the active region to the localized surface plasmon resonance [24]. This effectively suppresses non-radiative recombination channels and increases the IQE. A coating of core/shell Ag/SiO_2_ NPs results in an even better stability under typical LED operating conditions. However, there is still a lack of research regarding the methods of fabrication, architectural control, and the physical properties of hybrid nanostructures based on III-N NWs decorated with Ag NPs. 

There is a large variety of possible design schemes for fabricating hybrid metal/semiconductor structures to allow the observation of phenomena yet to be explored or understood. GaN nanostructures are known for their high structural quality, but for InGaN, the growth process becomes more difficult [25]. The main reason for this problem is the degradation of crystalline quality due to an increase in lattice mismatch between InGaN and the underlying GaN. The higher the indium content, the poorer the structural quality, leading to an increased density of threading dislocations that act as non-radiative recombination channels. This is a critical issue in In-rich InGaN-based LEDs and results in a reduced efficiency when operating in the green region of the spectrum. Additionally, InGaN tends to phase-segregate into In-rich and Ga-rich alloy regions during growth, particularly as the indium fraction increases. This is due in part to the high surface mobility of In atoms compared to Ga atoms and a significant difference in the vapor pressures of these metals. Reducing the growth temperature to prevent the thermal segregation of InGaN is a challenge because the cracking efficiency of ammonia, a typical nitrogen precursor, sharply decreases at lower temperatures, resulting in low growth rates and poor crystal quality. As a result, the luminescence of InGaN is known to be much broader and weaker, which further complicates its use in research.

Planar microcavities are widely used in the field of optoelectronics to enhance the spontaneous emission rate. In planar GaN NWs, the light–matter interaction can lead to the appearance of pronounced cavity modes [11,12]. Planar NW design offers advantages such as a high surface-to-volume ratio, a large surface area for efficient light extraction, and easy integration with other devices. Thus, in this work, we focused on studies of hybrid structures fabricated using plasmonic Ag NPs and high-quality planar GaN NWs. We observed a modification of the emission properties in the GaN NWs in the vicinity of Ag NPs and new recombination lines below the near-band gap emission in GaN. A theoretical model considering the Fröhlich resonance approximation is suggested to explain the enhancement of these emission lines.

## 2. Materials and Methods

Planar GaN NWs were grown at ~1000 °C by selective-area MOVPE. Trimethylgallium (TMG) and ammonia (NH_3_) were used as precursors for Ga and N, respectively. First, a 3 µm thick GaN layer was grown on (0001) sapphire substrates, and then a 5 nm thick Si_3_N_4_ layer was deposited, in which trenches with a 500 nm width were etched by FIB. For details of the etching, see [11]. After the FIB processing, the MOVPE overgrowth was carried out to form GaN NWs. The pattern on the layer consisted of several groups of planar NWs. To study the effect of metallic nanoparticles on the emission properties of GaN NWs, Ag nanoparticles were deposited on one group of GaN NWs using the drop casting technique. The Ag nanoparticles were purchased from Sigma-Aldrich (Sigma-Aldrich Sweden AB, Stockholm, Sweden) and have a spherical size of 60 nm according to TEM. As the size of the Ag NPs decreased, the optical density maximum was shifted to higher energies. The size of 60 nm was chosen because the maximum is in the range of 425–450 nm, and there is a significant overlap between the absorption spectra of Ag NPs and the emission spectrum of GaN. To ensure precise positioning of the NPs, we used scanning electron microscopy (SEM), which still allowed for the resolution of 60 nm nanoparticles.

For sample characterization we also used a MonoCL4 system (Gatan UK, Abingdon, UK) in situ SEM (Zeiss Sigma 300, Carl Zeiss Microscopy GmbH, Jena, Germany). Cathodoluminescence (CL) measurements were performed at a cryogenic temperature of 5 K with an acceleration voltage of 5 kV, allowing a spatial resolution of ~200 nm. Photoluminescence was measured using a micro-photoluminescence (µ-PL) set-up with a spatial resolution of ~1 µm. The third harmonic with a wavelength of λ_e_ = 266 nm from a 76 MHz Ti:sapphire femtosecond pulsed laser was used for PL excitation. The sample was placed into an Oxford Microstat (HiRes MK2, Oxford Instruments NanoScience, Abingdon, UK) to allow variable temperatures to be obtained between 5 and 295 K. Time-resolved PL (TRPL) measurements were done using a Hamamatsu syncroscan streak camera with a resolution of ~20 ps.

## 3. Results

**Figure 1:**
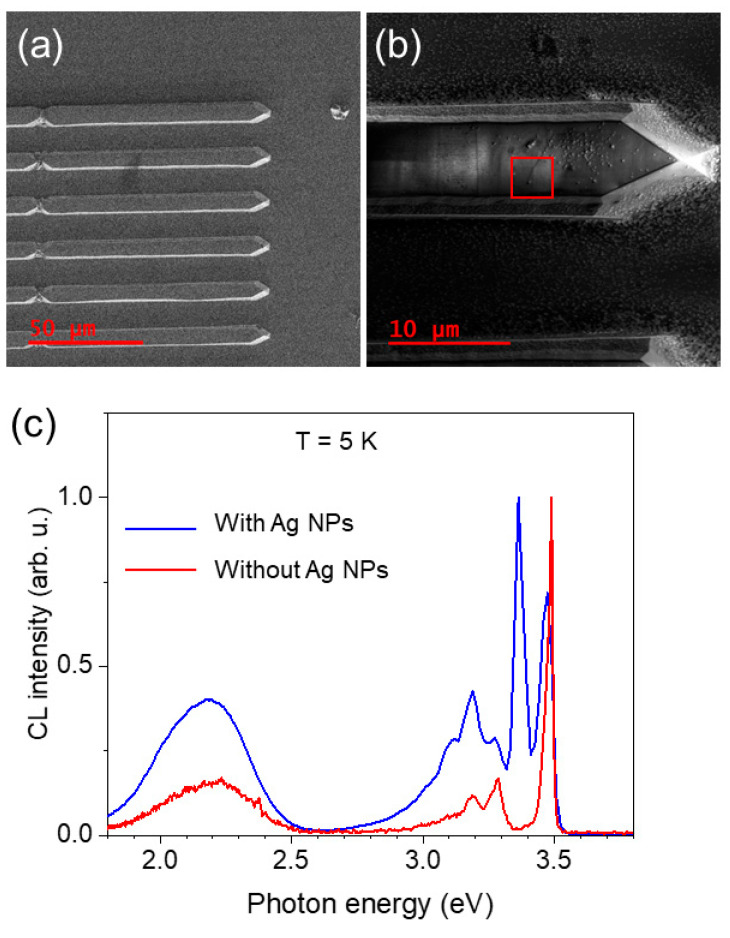
(**a**) Overview of SEM image of the planar GaN NWs without Ag NPs. (**b**) SEM image of one planar GaN NW with Ag NPs on the surface. The square indicates where the CL spectrum was measured. (**c**) Normalized CL spectra taken at 5 K for GaN NW without Ag NPs (red line) and with Ag NPs (blue line).

Figure 1a shows the SEM image of planar GaN NWs. Figure 1b shows a structure with deposited Ag NPs. Low-temperature emission properties have been studied for GaN NWs after the deposition of Ag NPs and, for comparison, for the GaN NWs without Ag NPs. Figure 1c shows a comparison of the CL spectra for the GaN structure without (red line) and with Ag NPs (blue line). The CL spectrum for planar NW without Ag NPs shows a typical emission for GaN with near-bandgap (NBG) excitonic emission at ~3.47 eV and donor–acceptor pair (DAP) recombination with a maximum at ~3.27 eV and its two LO phonon replicas [26]. After the deposition of Ag NPs, the CL spectrum changes as a new dominant line appears at ~3.36 eV. Additionally, the DAP emission shows a stronger line at ~3.20 eV instead of 3.27 eV. 

**Figure 2:**
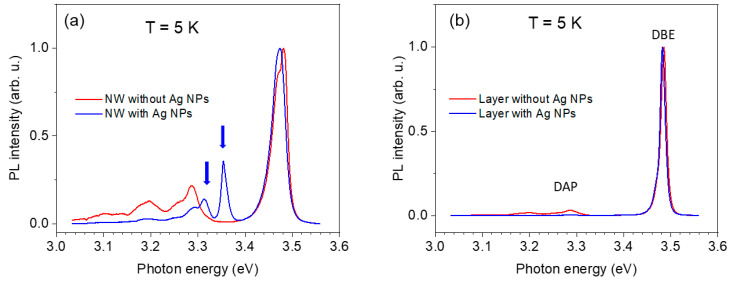
(**a**) Normalized time-integrated PL spectra taken at 5 K for bare GaN NW (red line) and for the hybrid Ag NPs/GaN NW structure. (**b**) Reference PL spectra taken at 5 K for the GaN layer without (red line) and with (blue line) Ag NPs.

The properties of the NBG emission of the hybrid Ag NPs/GaN NWs were studied by µ-PL and µ-TRPL. Typical time-integrated PL spectra at 5 K are shown in Figure 2a for the hybrid Ag NPs/GaN NW structure (blue line) and the bare GaN NW (red line). The PL spectra of the GaN NWs are similar to those of GaN epitaxial layers. At low temperatures (~5 K), the main NBG emission peak corresponds to the zero-phonon line of the exciton bound to shallow donors, such as silicon or oxygen impurities. If the background impurity level is very low and the lines have a full width at half maximum (FWHM) in the range of a few meV, different PL lines can be resolved, and the emission of the free exciton (zero-phonon line) can be observed as a weaker feature on the high-energy side of the PL line [27]. In our case, however, the FWHM of the DBE is ~30 and ~15 meV in GaN NWs and GaN layers, respectively, and the free exciton emission was not resolved. Similar to CL, a new line at ~3.36 eV appeared in the hybrid Ag NPs/GaN NWs structure. The FWHM of this line was estimated to be ~15 meV. Moreover, an additional new feature at ~3.31 eV can be seen at the shoulder of the DAP emission. A similar modification of the NBG emission was observed for several examined places on the hybrid structure. For reference, we measured the NBG emission from the GaN layer with and without Ag NPs (see Figure 2b). The PL spectra are almost identical and dominated by the donor-bound exciton (DBE) line at ~3.47 eV and weaker DAP emission, which is typical for the unintentionally doped GaN layers. Importantly, we did not observe new PL features for the GaN layer covered by the Ag NPs. 

**Figure 3:**
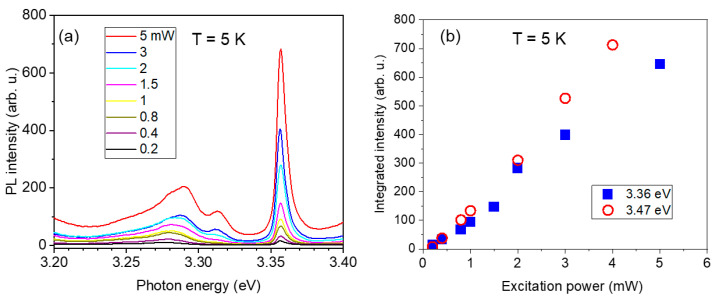
(**a**) Time-integrated PL spectra taken at 5 K at different excitation powers for the hybrid Ag NPs/GaN NW structure. Spectra are shown in the region around the 3.36 eV line. (**b**) Integrated PL intensity measured at 5 K for the 3.36 eV line (closed squares) and for the 3.47 eV DBE emission (open circles) vs excitation power.

The PL spectra in the hybrid Ag NPs/GaN NW structure were investigated depending on optical pumping. The low-temperature PL spectra in the region around a 3.36 eV line measured at different excitation powers are illustrated in Figure 3a. Figure 3b shows a power dependence of the integrated PL intensity for the 3.36 eV line (squares) and for the 3.47 eV DBE line (circles). Both lines show similar near-linear responses to the variation in the excitation power.

**Figure 4:**
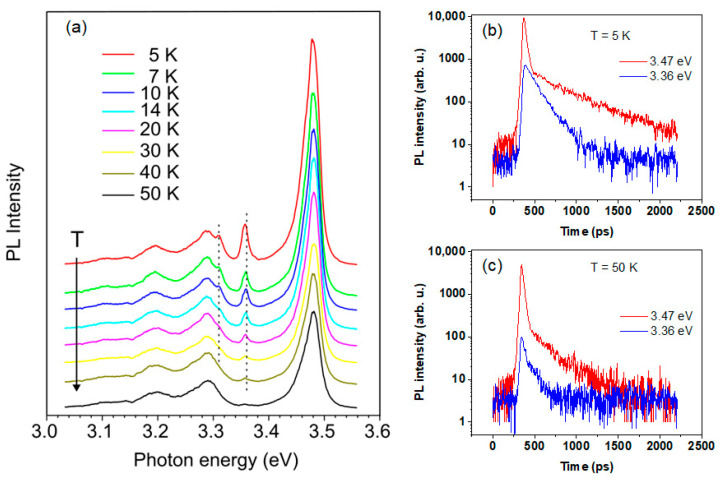
(**a**) Thermal evolution of PL spectra measured at an excitation power of 5 mW for hybrid Ag NPs/GaN NW structure. Spectra are shifted vertically for clarity. The dashed lines are a guide for the eye. PL decay curves at 5 K (**b**) and 50 K (**c**) for the hybrid Ag NPs/GaN NW structure measured at the DBE peak at 3.47 eV (red line) and at the 3.36 eV feature (blue line).

The temperature dependence of the PL spectra for the hybrid Ag NPs/GaN NWs are shown in Figure 4a. The emission at 3.36 eV vanished rather quickly with increasing temperature and was almost completely thermalized at 50 K, giving an activation energy of about 4.3 meV. The dynamic properties for the 3.36 eV line and for the DBE at 3.47 eV were also different, especially at a low temperature, as illustrated in Figure 4b. For the line at 3.47 eV, there are two processes with fast and slow decay with a PL lifetime of 30 and 260 ps, respectively. This indicated an overlap between the fast recombination related to the free exciton and slow emission related to the bound exciton. For the 3.36 eV line, the decay was single exponential with a recombination time of 120 ps. Figure 4c shows PL decay curves measured at 50 K, where the decay of the recombination at 3.47 eV still has the contributions of the fast and slow components with lifetimes of ~30 and 190 ps, respectively. For the 3.36 eV line, the weak emission shows a decay time of ~60 ps. Note that for the DAP recombination at 3.27 eV, the decay is very slow and exceeds 12 ns (i.e., the period between the laser pulses), while the feature at the shoulder (~3.31 eV) shows a much faster decay. Clearly, from the PL decay measurements, the line at 3.36 eV had a different origin compared to a free or bound exciton.

## 4. Discussion

To explain the enhancement of emission lines in the luminescence spectra of the hybrid Ag NPs/GaN NWs structure, we considered a theoretical model based on Fröhlich approximation. Light scattering by small particles is a long-standing fundamental problem in electrodynamics, the theory of which includes both near- and far-field descriptions. This light scattering by a small particle began with the consideration of the electric dipole concept and the assumption of constant electromagnetic phase within the region of interest, which is valid if the particle size is much smaller than the light wavelength. The polarization (i.e., the induced dipole) of materials caused by the electromagnetic field determined by the dielectric function results in the light scattering [28]. The solution for optical scattering by a sphere of any size in a homogeneous medium is known as the Mie theory and can also be applied to scattering using multiple spheres if the distance between them is large enough [29]. 

In our case, the size of the Ag NPs is ~60 nm, and their shape is almost spherical. This allows the NPs to be approximated as spheres with a radius *r* of 30 nm, which is much less than the wavelength λ in the medium (λ = 356 nm for GaN exciton at 5 K). When the radius of a nanoparticle is significantly smaller than the wavelength (*r* << λ), the scattering of light can be explained using the Fröhlich resonance approximation [28,30,31,32]. In the case of a metal nanoparticle in a dielectric medium, collective oscillations of free electrons in the metal lead to the appearance of surface modes, which manifest themselves in the features of the scattering and absorption spectra. The specific spectral position of these features is determined by the Fröhlich condition for the permittivity of the metal and the matrix. Today, hybrid materials formed by introducing resonant elements of subwavelength size into a certain matrix or onto the surface of a material play an important role in the development of photonics since they allow the modeling of the necessary electromagnetic response of the system to obtain the desired optical properties. For example, such materials are used in sensors, solar panels, antennas, and absorbers [33]. The Fröhlich resonance approximation makes it possible to describe the optical properties of such nanostructures accurately, as was shown previously [34,35,36].

The electric field of the light induces polarization, and a small particle can be considered a radiative dipole. In this case, a resonant feature in the spectra of spherical metal nanoparticles is expected to occur near the wavelength that satisfies the Fröhlich condition:

(1) $$Re\left\{\varepsilon \left(\omega \right)\right\}=-2Re\left\{\varepsilon_{m}\left(\omega \right)\right\}$$

where ε and $\varepsilon_{m}$ are the dielectric constant of the metal and the medium, respectively. The effect of the Fröhlich resonance of metal nanoparticles on the optical spectra of photonic structures has been described earlier [34,37,38]. In the general case, as aforementioned, the scattering of spherical particles is described using the Mie theory, which gives the scattering and extinction cross-sections as a sum of the electric and magnetic spherical waves (harmonics). Dipole scattering describing the Fröhlich resonance is given by the electric harmonic with the quantum number *l* = 1 [28,30].

For a particle made of pure silver, the polarizability *α* can be written as:

(2) $$\alpha =r^{3}\cdot \frac{\varepsilon -\varepsilon_{m}}{\varepsilon +2\varepsilon_{m}}$$

The scattering cross-section is expressed as:

(3) $$C_{scat}=\frac{k^{4}}{6\pi }{\left|\alpha \right|}^{2\;},$$

where *k* is the wave vector in the medium. The dielectric constant for silver was calculated according to the Drude model. The scattering cross-section spectrum is an important quantity because the strength of the electric field localized near the surface of a metal nanoparticle on a semiconductor nanowire is proportional to the scattering cross-section on that nanoparticle. Hence, there is a correlation between a feature in the scattering cross-section spectrum and the PL spectrum of nanowires [28,38].

**Figure 5:**
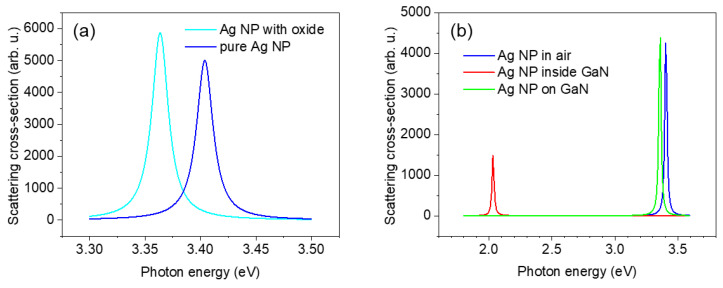
(**a**) The scattering cross-section spectra calculated for a bare silver nanoparticle in air (blue line) and for the Ag NP covered with a 1.5 nm thin oxide film (cyan line). (**b**) The scattering cross-section spectra are compared for the Ag NP in air (blue line), embedded in GaN (red line), and placed on the GaN surface with contact area of 10 % (green line).

Below we consider different approaches. First, a small NP consists of pure Ag and has no contact with GaN; the medium in this case is air. The scattering cross-section spectrum calculated according to Equation (3) is depicted in Figure 5a,b by the blue line. The peak resonance position of a bare Ag NP corresponds to 3.42 eV, which is shifted to the blue region compared to the experimentally observed peak at 3.36 eV. Note that the calculations for particles in the range of 50–70 nm are not affected by the NP size on the position of the plasmonic resonance. However, if the Ag NP is oxidized, then the resonance condition for a metal particle covered with a dielectric shell is given by the formula [38]:

(4) (Re{ε2ω}+2Reεmω)(Re{ε1ω}+2Reεmω)=−2rinrout3(Re{ε2ω}−Reεmω)(Re{ε1ω}−Re{ε2ω})  ,

where $\varepsilon_{1}$ is the dielectric constant of the metal; $\varepsilon_{2}$ is the dielectric constant of the shell; $r_{in}$ is the metal core radius; and $r_{out}$ is the radius of the entire sphere. In this case, the polarizability can be written as:

(5) α=rout3·(ε2−εm)ε1+2ε2+rinrout3(ε1−ε2)εm+2ε2(ε2+2εm)(ε1+2εm)+2rinrout3(ε2−εm)(ε1−ε2)

Calculations using Equation (5) for a metal–dielectric core-shell structure with an inner and outer radius of 30 and 31.5 nm, respectively, show that the resonance is shifted to lower energies, as represented by the cyan line in Figure 5a. The refractive index for silver oxide from 3.0 to 3.5 eV was taken to 1.2 [39]. Though this approach is in line with the experimental position of the 3.36 eV peak, there are doubts that silver particles can be easily oxidized under normal media conditions. 

A more likely explanation for the observed phenomenon is the effective medium approach. The Ag NP has a non-zero contact area with the GaN NW surface. Using Equation (2), the spectra of the scattering cross-section were calculated for a silver nanoparticle immersed in various media such as air, GaN, and an effective medium consisting of air and GaN (Figure 5b). The dispersion data for the refractive index of wurtzite GaN were taken from [40]. Clearly, the presence of the GaN substrate shifts the position of the resonance to the low-energy region. The effective medium consisting of air and containing 10% GaN describes the experimental results well, as shown by the peak at 3.36 eV (Figure 5b, green line). Although the size of the small sphere had almost no influence on the resonance frequency of bare Ag NPs, the size of the contact region with GaN affected the refractive index of the effective medium and hence the position of the resonant peak, which explains the more complex modification of the experimental emission spectra near Ag NPs. Note that the experimental width of the 3.36 eV line agrees with the spectral width (FWHM = ~17 meV) for the Fröhlich resonance. 

The effective medium approach also explains the absence of the Fröhlich resonance effect in the case of the GaN layer. Due to a remaining mask film, the resonance frequency of the plasmonic Ag NP placed on the Si_3_N_4_ mask layer will be shifted to the energy region below 3 eV, and no enhancement of the PL emission can be observed.

In the case of hybrid structures based on InGaN NWs and Ag NPs, there was also a modification of the PL spectra and a moderate enhancement of emission at the position that corresponds to the Fröhlich resonance conditions in small Ag NPs [38]. However, due to the broad emission of the InGaN NWs caused by the inhomogeneity of In, which results in a refractive index difference of ~0.1, the resulting PL line enhancement was not so obvious due to its much larger spectral width compared to the Fröhlich resonance. In our case of pure GaN structures, there is no deviation in the refractive index, and the enhanced PL feature at 3.36 eV has nearly the same width as the theoretical line.

## 5. Conclusions

We studied the optical properties of hybrid structures formed by Ag NPs and GaN planar NWs grown using a selective area MOVPE process. The growth involved depositing a 3 µm thick GaN layer on sapphire substrates, followed by the deposition of a 5 nm thick mask layer where trenches were etched using FIB. After FIB processing, MOVPE overgrowth was performed to form GaN NWs. To study the effect of metallic nanoparticles on the emission properties, silver NPs of a spherical shape with a radius of 30 nm were deposited on GaN planar NWs. 

Measurements of the PL spectra of the GaN NWs using the µ-PL setup were similar to those of the GaN epitaxial layers. In the case of the hybrid Ag NPs/GaN NWs structure, at low temperatures, a new narrow line with an FWHM of 15 meV appeared at ~3.36 eV in the vicinity of Ag NPs. However, the PL spectra from the GaN layer with and without Ag NPs were almost identical, and no new PL features were observed for the GaN layer covered by the Ag NPs. 

A theoretical model based on Fröhlich resonance approximation was suggested. The scattering cross-section spectrum was calculated for a bare Ag NP in air, resulting in the peak position of the resonance of 3.42 eV. To obtain a correlation with the experiment, we also considered an oxidized Ag NP and the effective medium approach. The latter approach was adopted because the Ag NP had a non-zero contact area with the GaN NW surface. The presence of the GaN substrate shifted the position of the resonance to the low-energy region, and the effective medium consisting of air and 10% GaN described the experimental results well.

## Data Availability

The data presented in this study are available on reasonable request from the corresponding author.
